# Optimization of soybean physiochemical, agronomic, and genetic responses under varying regimes of day and night temperatures

**DOI:** 10.3389/fpls.2023.1332414

**Published:** 2024-02-06

**Authors:** Chuanbo Ding, Fahad Alghabari, Muhammad Rauf, Ting Zhao, Muhammad Matloob Javed, Rahma Alshamrani, Abdel-Halim Ghazy, Abdullah A. Al-Doss, Taimoor Khalid, Seung Hwan Yang, Zahid Hussain Shah

**Affiliations:** ^1^ College of Traditional Chinese Medicine, Jilin Agriculture Science and Technology College, Jilin, China; ^2^ Department of Plant Breeding and Genetics, Pir Mehr Ali Shah, Arid Agriculture University, Rawalpindi, Pakistan; ^3^ Department of Plant Production, College of Food and Agriculture Science, King Saud University, Riyadh, Saudi Arabia; ^4^ Department of Biotechnology, Chonnam National University, Yeosu, Republic of Korea

**Keywords:** antioxidant, correlogram, gene expression, heat stress, principal component analysis, soybean

## Abstract

Soybean is an important oilseed crop worldwide; however, it has a high sensitivity to temperature variation, particularly at the vegetative stage to the pod-filling stage. Temperature change affects physiochemical and genetic traits regulating the soybean agronomic yield. In this regard, the current study aimed to comparatively evaluate the effects of varying regimes of day and night temperatures (T1 = 20°C/12°C, T2 = 25°C/17°C, T3 = 30°C/22°C, T4 = 35°C/27°C, and T5 = 40°C/32°C) on physiological (chlorophyll, photosynthesis, stomatal conductance, transpiration, and membrane damage) biochemical (proline and antioxidant enzymes), genetic (*GmDNJ1*, *GmDREB1G;1*, *GmHSF-34*, *GmPYL21*, *GmPIF4b*, *GmPIP1;6*, *GmGBP1*, *GmHsp90A2*, *GmTIP2;6*, and *GmEF8*), and agronomic traits (pods per plant, seeds per plant, pod weight per plant, and seed yield per plant) of soybean cultivars (Swat-84 and NARC-1). The experiment was performed in soil plant atmosphere research (SPAR) units using two factorial arrangements with cultivars as one factor and temperature treatments as another factor. A significant increase in physiological, biochemical, and agronomic traits with increased gene expression was observed in both soybean cultivars at T4 (35°C/27°C) as compared to below and above regimes of temperatures. Additionally, it was established by correlation, principal component analysis (PCA), and heatmap analysis that the nature of soybean cultivars and the type of temperature treatments have a significant impact on the paired association of agronomic and biochemical traits, which in turn affects agronomic productivity. Furthermore, at corresponding temperature regimes, the expression of the genes matched the expression of physiochemical traits. The current study has demonstrated through extensive physiochemical, genetic, and biochemical analyses that the ideal day and night temperature for soybeans is T4 (35°C/27°C), with a small variation having a significant impact on productivity from the vegetative stage to the grain-filling stage.

## 1 Introduction

Soybean (*Glycine max* L.) is an important legume crop providing approximately 29% of the oil and 71% of protein for humans and livestock in the world (Alsajri et al., 2022). With the sudden rise of the world population, there is a need to keep the pace of soybean production compatible with human demand under changing climatic conditions (Raza et al., 2019). Among projected climatic changes, the temperature is reported to have more adverse effects on main crops, such as soybeans (Osei et al., 2023). The average annual temperatures of regions producing wheat, rice, corn, and soybean have increased by 1°C during the past century (Zhao et al., 2017). Climate modeling experts forecast that the 21st century will witness a rise of temperatures by 1°C–4°C depending upon the region (Fahad et al., 2017). The coincidence of high temperature with flowering and grain-filling stages causes a severe reduction in yield due to the impairment of physiological processes (Alsajri et al., 2019). Furthermore, high temperatures trigger oxidative stress due to the generation of reactive oxygen species (ROS) (Sachdev et al., 2021). Plants like other living systems tend to retain their homeostatic balance (Awasthi et al., 2015). Therefore, plants activate ROS scavenging mechanisms by enhancing the activities of antioxidant enzymes such as peroxidase (POD), catalase (CAT), and superoxide dismutase (SOD) (Rajput et al., 2021). Additionally, a high concentration of ROS impairs the structural integrity of the cell membrane, resulting in membrane damage and less water retentively due to more electrolyte leakage (Jianing et al., 2022). With every 0.8°C rise above the mean temperature, soybean yield is projected to decrease by 2.4% (Djanaguiraman et al., 2019). Hence, it is mandatory to understand the responses of soybeans to temperature variations for devising mitigation strategies. Although a rise in temperature impacts adversely the soybean reproductive phase and seed formation, the effect of temperature changes varies with extent, the period, and cultivars (Djanaguiraman et al., 2019). It has been projected that soybean production decreased by 17% with every 1°C rise in temperature above optimum in soybean-growing regions (Yang et al., 2023). In addition, the rise of temperature above optimum significantly decreases agronomic yield such as pods per plant, seed size, seed number, and seed yield in soybeans (Choi et al., 2016). Likewise, reproductive and grain-filling stages, biochemical, and physiological activities are also highly susceptible to high temperatures. Photosynthesis is among the primary cell events that are highly prone to high-temperature stress and are impaired on priority before the inhibition of other events (Mathur et al., 2014). The foremost target site of high temperature is photosystem II (PSII), which is an integral part of chlorophyll (Sasi et al., 2018). Therefore, reduction in chlorophyll content and disruption of chloroplast function lead to decreased photosynthesis and crop productivity. With every 4°C rise in temperature up to optimum, soybean shows a 59% increase in net photosynthesis (Alsajri et al., 2022). Additionally, there is a 17% drop in net photosynthesis when the temperature is raised by the same percentage above the optimal level (Ortiz et al., 2022). Plant responses to environmental stresses are genetically regulated; therefore, it is important to elucidate the relative expression of temperature stress-associated genes under varying regimes of temperatures. Heat shock proteins (HSPs) play an essential role in providing tolerance against biotic and abiotic stresses. In addition, HSPs increase the scavenging of ROS by positively regulating the antioxidant enzymes and enhancing membrane stability (Ul-Haq et al., 2019). For instance, Huang et al. (2019) noticed that the overexpression of soybean gene *GmHsp90A2* under high temperatures is associated with a decrease in oxidative stress and high chlorophyll content. Similarly, *GmDNJ1*, a type of HSP-40, also regulates the antioxidant enzymes and chlorophyll under heat stress as reported by Li et al. (2021). The dehydration-responsive element-binding protein (DREB) regulates the expression of the pyrabactin resistance 1-like (PYL) gene that enables plants to retain normal physiological and biochemical activities under the increasing regime of temperatures (Kidokoro et al., 2015). Moreover, Di et al. (2018) identified 14 PYL genes in *Brassica napus* that play vital roles in ABA signaling during different regimes of heat stress. In fact, heat stress raises endogenous ABA content that keeps water balanced and increases heat tolerance by regulating stomatal conductance (Hsieh et al., 2013). Additionally, the phytochrome interacting factor 4 (PIF4) mediates physiological and molecular processes in soybean by regulating the HSPs and transcripts of heat shock factors (HSP) (Arya et al., 2023). The HSF proteins increase plants’ endurance to heat stress and enable plants to retain their essential metabolic activities under heat stress (Li et al., 2014). The overexpression of soybean plasma membrane intrinsic protein 1;6 (*GmPIP1;6*) during heat stress optimizes the physiological processes and agronomic yield (Zhou et al., 2014). Moreover, tonoplast intrinsic proteins (TIPs) are also responsible for regulating the movement of water and the molecules of physiological significance; hence, they facilitate plants to sustain essential physio-chemical processes during abiotic stress (Kapilan et al., 2018). Likewise, in another study, Feng et al. (2019) reported that gene *GmTIP2;6* enhances plant growth under heat stress by modulating the activities of some essential proteins. Correspondingly, the gibberellic acid myeloblastosis (GAMYB)-binding protein (GBP) is an important gene of the GA pathway and encodes a gibberellin-induced regulatory protein that is involved in plant reproductive development (Bienias et al., 2020). Moreover, GBP plays a vital role in plant growth, cell differentiation, physiological processes, secondary metabolism, and tolerance to abiotic stress (Zhang et al., 2020). For instance, the gene *GmGBP1* depicts positive upregulation with the increasing regime of heat stress, enables tolerance to heat stress, and sustains soybean normal growth activities (Zhao et al., 2013). The elongation factor (EF) gene *GmEF8* enhances protein levels when soybean faces temperature stress and has a protective role *via* osmotic adjustments (Zhang et al., 2022). Soybean plants with high tolerance to temperature stress show a high transcript level of *GmEF8* with high proline content as compared to plants grown under control conditions (Jianing et al., 2022). The understanding of genetic responses under varying levels of temperature provides a foundation to further understand the temperature response pathway (Jianing et al., 2022). Tariq et al. (2022) performed transcriptome analysis of soybean genotypes NARC-1 and Swat-84 under the same conditions within a glass house; however, the detailed genetic analysis in association with physiological, biochemical, and morphological traits is lacking. Additionally, soybean is a temperature-sensitive crop; therefore, we comprehensively evaluated soybean genotypes at varying regimes of day and night temperatures by focusing on physiological, biochemical, agronomic, and genetic indices. Temperature directly affects the genetic, physiological, and biochemical traits of soybeans, which are ultimate determinants of agronomic productivity. In this context, the current study aimed to investigate the impacts of varying regimes of temperature on the genetic, physiological, biochemical, and agronomic traits of soybean cultivars (NARC-1 and Swat-84). Furthermore, the current study aimed to know how physiological, biochemical, and genetic traits are interconnected to determine the agronomic productivity of soybean cultivars.

## 2 Materials and methods

The present study was performed in soil plant atmosphere research (SPAR) units located at the experimental area of Jilin Agricultural Science and Technology Center, Jilin, China. Two soybean cultivars, the thermosensitive Swat-84, released in 1984 by the Agricultural Research Institute, Swat Pakistan (Asad et al., 2020), and the thermotolerant NARC-1, released in 1991 by National Agricultural Research Center, Islamabad, Pakistan (Asad et al., 2020), were evaluated at five different regimes of day/night temperatures (T1 = 20°C/12°C, T2 = 25°C/17°C, T3 = 30°C/22°C, T4 = 35°C/27°C, and T5 = 40°C/32°C), which were obtained *via* thermo-static adjustments of the five SPAR units (Alsajri et al., 2020). The tri-replicate experiment was conducted in a two-factorial design, with cultivars as one factor and temperature as the other factor.

### 2.1 Plant growth and temperature treatment

The temperature during the day was applied at sunrise, while the temperature during the night was applied after sunset. Seeds were sown at 2-cm depth in a plastic container having a diameter of 30 cm and a height of 50 cm. The pots were supplemented with gravel at the bottom and filled with a 3:1 mixture of topsoil and sand. Additionally, plants were fertilized with Hoagland nutrient solution using an automated drip irrigation system every day at 7:00 a.m., 12:00 a.m., and 5 p.m. Furthermore, solar radiations were recorded regularly using a pyranometer. Moreover, temperature treatments were applied starting at the vegetative stage V3-Vn (Purcell et al., 2014) and continued till the seed-setting stage R5 (Purcell et al., 2014). For each treatment in a replicate, five pots each containing three plants were used.

### 2.2 Physiological quantification

The chlorophyll (Chl) content was determined using the SPAD-502Plus (Konica Minolta, Langenhagen, Germany) from three different leaves of each plant, and data were recorded as average. Furthermore, the IRGA apparatus (ADC Bioscientific, Hoddesdon, UK) was used to record stomatal conductance (Gs), photosynthesis rate (Pn), and transpiration rate (Tr) from soybean leaves between 8:00 a.m. and 10:00 a.m. Moreover, the cell membrane damage (MD) was measured following the procedure used by Sairam et al. (1997). To measure MD, two test tubes each with 20 mL of deionized water were used, and 100 mg of soybean leaf pieces was placed in each tube. One tube was put in the water bath at 40°C for 30 min to record conductivity A, and a second tube was put in a water bath at 100°C for 10 min to record conductivity B. Afterward, the MD was calculated using relation [1 − (A/B)] × 100. For physiological assessments, the data for each treatment were collected from randomly selected five plants on a weekly basis from the start to the end of treatment. The data for each treatment were averaged for statistical analysis. Additionally, the results showing the significant variation at the R5 stage were only included for analysis.

### 2.3 Biochemical quantification

For the assay of antioxidant enzymes, 1 g of frozen leaves was homogenously mixed in 1 mL of ice-cold 0.1 M Tris-HCl buffer with pH 7.4. Afterward, the mixture was centrifuged at 20,000 *g* and 4°C for 15 min, and the supernatant was extracted to record the enzymatic activity following the procedure opted by Djanaguiraman et al. (2018). The SOD activity was estimated using the SOD assay kit (Cell Biolabs, San Diego, CA, USA) following the instructions provided by the manufacturer. Correspondingly, the CAT activity was estimated using the CAT assay kit (Cell Biolabs, USA) according to the manufacturer’s instructions. Likewise, the POD assay kit (Cell Biolabs, USA) was used for the estimation of POD activity following the manufacturer’s protocol. Meanwhile, proline content was measured using a UV-Vis spectrophotometer (Konica Minolta, Langenhagen, Germany) based on ninhydrin reactivity. For the measurement of biochemical traits, the data for each treatment in a replicate were taken from randomly selected five plants on a weekly basis from the start till the end of temperature treatments. Afterward, the data for each treatment were averaged for analysis. Additionally, the results illustrating significant variation at the R5 stage were only included in the analysis.

### 2.4 Measurement of agronomic characters

The agronomic characters, pods per plant (PPP), and seeds per plant (SPP) from randomly selected five plants of each treatment were counted and averaged for statistical analysis. Correspondingly, pod weight per plant (PWPP) and seed yield per plant (SYPP) from randomly selected plants of each treatment were measured using weighing balance and averaged afterward for statistical analysis.

### 2.5 Gene relative expression analysis

The genes *GmDNJ1*, *GmDREB1*, *GmHSF-34*, *GmPYL21*, *GmPIF4b*, *GmPIP1;6*, *GmGBP1*, *GmHsp90A2*, *GmTIP2;6*, and *GmEF8* were relatively analyzed at R5 stage for their expression. For relative gene expression, RNA was extracted from selected leaf samples of soybean genotypes (Swat-84 and NARC-1) exposed to varying regimes of day and night temperatures (T1 = 20°C/12°C, T2 = 25°C/17°C, T3 = 30°C/22°C, T4 = 35°C/27°C, and T5 = 40°C/32°C) by using an RNA extraction kit (Cell Biolabs, USA). Afterward, the cDNA library was established according to Ding et al. (2020). For this purpose, 2 μg of RNA sample was used, and qRT-PCR was performed according to the cited procedure. In addition, the relative gene expression was normalized by the *GmActin* gene. The sequences of primers are indicated in **Table 1**.

**Table 1:** List of primers used for relative expression of genes under changing regimes of temperatures.

Gene	Accession no.	Primer sequence
*GmDNJ1*	Glyma.12G095700	TAAGACATCTTGGCCCATCC (F) CACAACCTTCTCTCCCTTGC (R)
*GmDREB1G;1*	Glyma.14G084700	CAACTCCAAAGGGAGGGTTCC (F) CAAAAGAACCTTTCAGAACCTCCTTC (R)
*GmPYL21*	Glyma.13g29380	TGAGGTGGTTTCAAGCTGTCA (F) GCCTACAAAGGAATCGAATCAATC (R)
*GmHSF-34*	Glyma.17g34540	ACTTACAGAAGGCACAGAGGA (F) ACACTTGTTTCAGTTCAGGGA (R)
*GmPIF4b*	Glyma.14G032200.1	CTGTGGCAGCAGTCATATCC (F) TCTGATTTTCCTTTGTCACTCC (R)
*GmPIP1;6*	Glyma.08G015300	AACTATGAGTTGTTCAAAGGA (F) AGAAAACGGTGTAGACAAGAAC (R)
*GmHsp90A2*	Glyma.14g40320	CTGTTTTGTGTTCTAACAATGGCT (F) GATTTGTAACTTATTCTATGAGGGCA (R)
*GmGBP1*	Glyma.01G008600v4	TGAGAAATAAAAGTGGATAGGAAAAG (F) TGGAAGATATAATATATGAGGGAGGA (R)
*GmTIP2;6*	Glyma.15G018100	CACTGGCTATGACACTCCTATTC (F) ACACCGTGTACACTAATCCAAA (R)
*GmEF8*	Glyma.19G052400	GGCTGATTGTGCTGTCCTT (F) GGTAGTGGCATCCATCTTGTTA (R)

### 2.6 Data analysis

Analysis of variance (ANOVA) at a 5% probability level was applied to analyze the data statistically. For this purpose, Statistix ver. 8.1 (McGraw-Hill, 2008) was used. Furthermore, correlation, principal component analysis (PCA), and heatmap cluster analysis were carried out using RStudio version 1.1.456 (RStudio Team, 2020). For PCA, “factoextra” and “FactoMineR” R packages were used. Pearson’s correlation was performed using R packages “GGally” and “ggplot2”, and heatmap cluster analysis was performed using “pheatmap” and complex Heatmap R packages.

## 3 Results

### 3.1 Physiological traits

**Figure 1:**
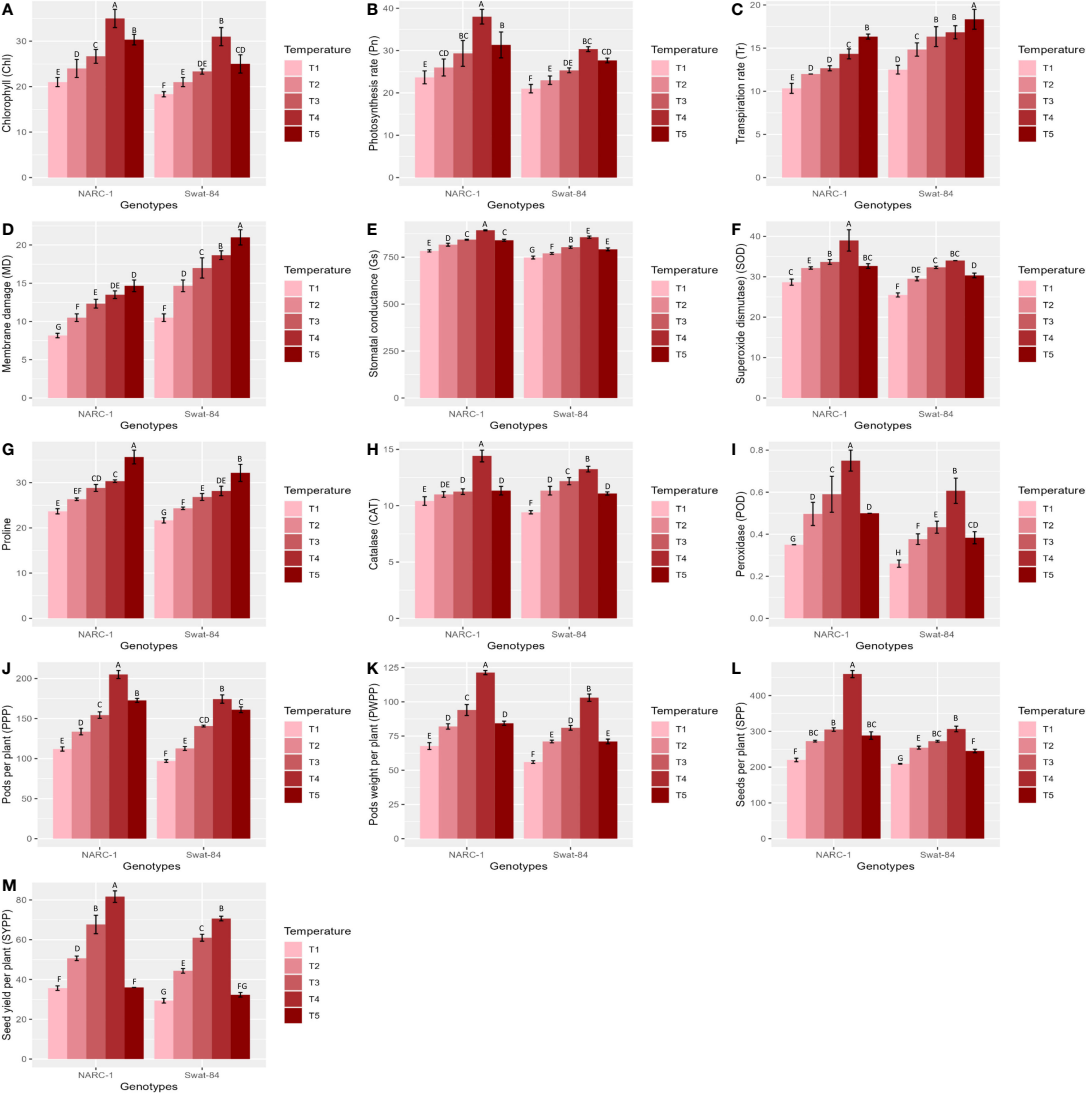
**(A**–**M)** Effect of varying regimes of day and night temperatures on physiological, biochemical, and agronomic traits of soybean cultivars. Chl, chlorophyll; CAT, catalase; Gs, stomatal conductance; MD, membrane damage; Pn, photosynthesis; POD, peroxidase; PPP, pods per plant; PWPP, pods weight per plant; SOD, superoxide dismutase; SPP, seeds per plant; SYPP, seed yield per plant; Tr, transpiration rate. T1 = 20°C/12°C, T2 = 25°C/17°C, T3 = 30°C/22°C, T4 = 35°C/27°C, and T5 = 40°C/32°C. Values in figures are mean estimates averaged at R5 stage of soybean development, bars show standard deviation ( ± SD), and letters represent significant differences at p ≤ 0.05. Units: Gs (mmol m^−2^ s^−1^); Pn (µmol m^−2^ s^−1^); Tr (mmol m^−2^ s^−1^); Chl (g/kg); POD, CAT, and CAT activities (enzyme units); proline (μg/g FW); MD (%); PWPP (g); SYPP (g).

All physiological traits including chlorophyll (Chl), stomatal conductance (Gs), photosynthesis rate (Pn), transpiration rate (Tr), and MD varied significantly (p ≤ 0.05) with varying regimes of day and night temperatures (**Figure 1**). The chlorophyll (Chl), photosynthesis rate (Pn), and stomatal conductance (Gs) illustrated a steady increase from temperature regimes T1 (20°C/12°C) to T4 (35°C/27°C) with a sudden decrease at regime T5 in both soybean cultivars Swat-84 and NARC-1 (**Figure 1**). However, at the same regimes of day and night temperatures, NARC-1 recorded higher Chl, Pn, and Gs values as compared to Swat-84 (**Figure 1**). Moreover, at T1 (20°C/12°C), these traits showed minimum mean values in Swat-84 (Chl = 16 g/kg, Gs = 700 mmol m^−2^ s^−1^, and Pn = 20 µmol m^−2^ s^−1^) and NARC-1 (Chl = 20 g/kg, Gs = 750 mmol m^−2^ s^−1^, and Pn = 25 µmol m^−2^ s^−1^) and maximum mean values at T4 (35°C/27°C) in both Swat-84 (Chl = 30 g/kg, Gs = 800 mmol m^−2^ s^−1^, and Pn = 30 µmol m^−2^ s^−1^) and NARC-1 (Chl = 35 g/kg, Gs = 850 mmol m^−2^ s^−1^, and Pn = 38 µmol m^−2^ s^−1^). In contrast, Tr and MD depicted consistently dramatic increases in both cultivars with increasing day and night temperature regimes T1 (20°C/12°C) to T5 (40°C/32°C) (**Figure 1**). Additionally, MD and Tr depicted minimum values at T1 (20°C/12°C) in Swat-84 (MD = 10% and Tr = 13 mmol m^−2^ s^−1^) and NARC-1 (MD = 8% and Tr = 10 mmol m^−2^ s^−1^) and maximum mean values in both Swat-84 (MD = 20%, Tr = 19 mmol m^−2^ s^−1^) and NARC-1 (MD = 15% and Tr = 17 mmol m^−2^ s^−1^) at T5 (40°C/32°C). Contrary to Chl, Pn, and Gs, the Tr and MD recorded higher values in Swat-84 as compared to NARC-1 at corresponding regimes of day and night temperatures (**Figure 1**).

### 3.2 Biochemical traits

The activity of all biochemical traits including antioxidant enzymes (SOD, CAT, and POD) and proline varied significantly (p ≤ 0.05) under varying regimes of day and night temperatures (**Figure 1**). The activity of antioxidant enzymes in terms of enzyme unit increased consistently from T1 (20°C/12°C) to T4 (35°C/27°C) and decreased afterward at T5 (40°C/32°C) in both cultivars (**Figure 1**). However, as compared to Swat-84, the cultivar NARC-1 revealed the maximum activities of antioxidant enzymes at the same regimes of temperatures (**Figure 1**). Furthermore, at T1 (20°C/12°C), the enzymes SOD, CAT, and POD illustrated the minimum activities in both genotypes Swat-84 (SOD = 28, CAT = 8, and POD = 0.2) and NARC-1 (SOD = 28, CAT = 8, and POD = 0.4) and the maximum mean values at T4 (35°C/27°C) in both Swat-84 (SOD = 35, CAT = 12, and POD = 0.6) and NARC-1 (SOD = 39, CAT = 14, and POD = 0.7). However, the concentration of proline depicted a significant (p ≤ 0.05) consistent increase in both cultivars with increasing regimes of temperatures from T1 (20°C/12°C) to T5 (40°C/32°C) (**Figure 1**). Moreover, at corresponding regimes of temperature, the soybean cultivar NARC-1 showed higher proline content as compared to Swat-84 (**Figure 1**). Additionally, proline showed the minimum value at T1 (20°C/12°C) in Swat-84 (proline = 20 μg/g FW) and NARC-1 (proline = 25 μg/g FW) and the maximum mean value in both Swat-84 (proline = 30 μg/g FW) and NARC-1 (proline = 35 μg/g FW) at T5 (40°C/32°C).

### 3.3 Agronomic traits

All agronomic traits such as PPP, PWPP, SPP, and SYPP varied significantly (p ≤ 0.05) due to varying regimes of day and night temperatures (**Figure 1**). All agronomic traits manifested a consistent increase in both soybean cultivars from temperature regimes T1 (20°C/12°C) to T4 (35°C/27°C) with a sudden decline at T5 (40°C/32°C) (**Figure 1**). However, under corresponding regimes of temperatures, NARC-1 showed comparatively high values of PPP, PWPP, SPP, and SYPP as compared to Swat-84 (**Figure 1**). Additionally, at T1 (20°C/12°C), the agronomic traits recorded minimum values in soybean genotypes, Swat-84 (PPP = 90, PWPP = 50 g, SPP = 200, and SYPP = 30 g) and NARC-1 (PPP = 110, PWPP = 70 g, SPP = 210, and SYPP = 38 g) and maximum mean values at T4 (35°C/27°C) in Swat-84 (PPP = 180, PWPP = 105 g, SPP = 310, and SYPP = 60 g) and NARC-1 (PPP = 210, PWPP = 125 g, SPP = 450, and SYPP = 85 g).

### 3.4 Correlation, principal component analysis, and heatmap analysis

**Figure 2:**
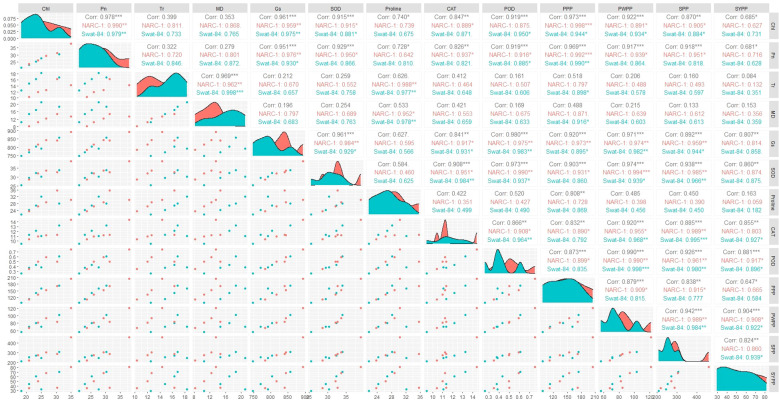
Correlogram showing the extent of overall paired association between physiological, biochemical, and agronomic traits in soybean cultivars due to varying regimes of temperature. Chl, chlorophyll; CAT, catalase; Gs, stomatal conductance; MD, membrane damage; Pn, photosynthesis; POD, peroxidase; PPP, pods per plant; PWPP, pods weight per plant; SOD, superoxide dismutase; SPP, seeds per plant; SYPP, seed yield per plant; Tr, transpiration rate. ***, Significant at p ≤ 0.001; **, Significant at p ≤ 0.01; *, Significant at p ≤ 0.05.

**Figure 3:**
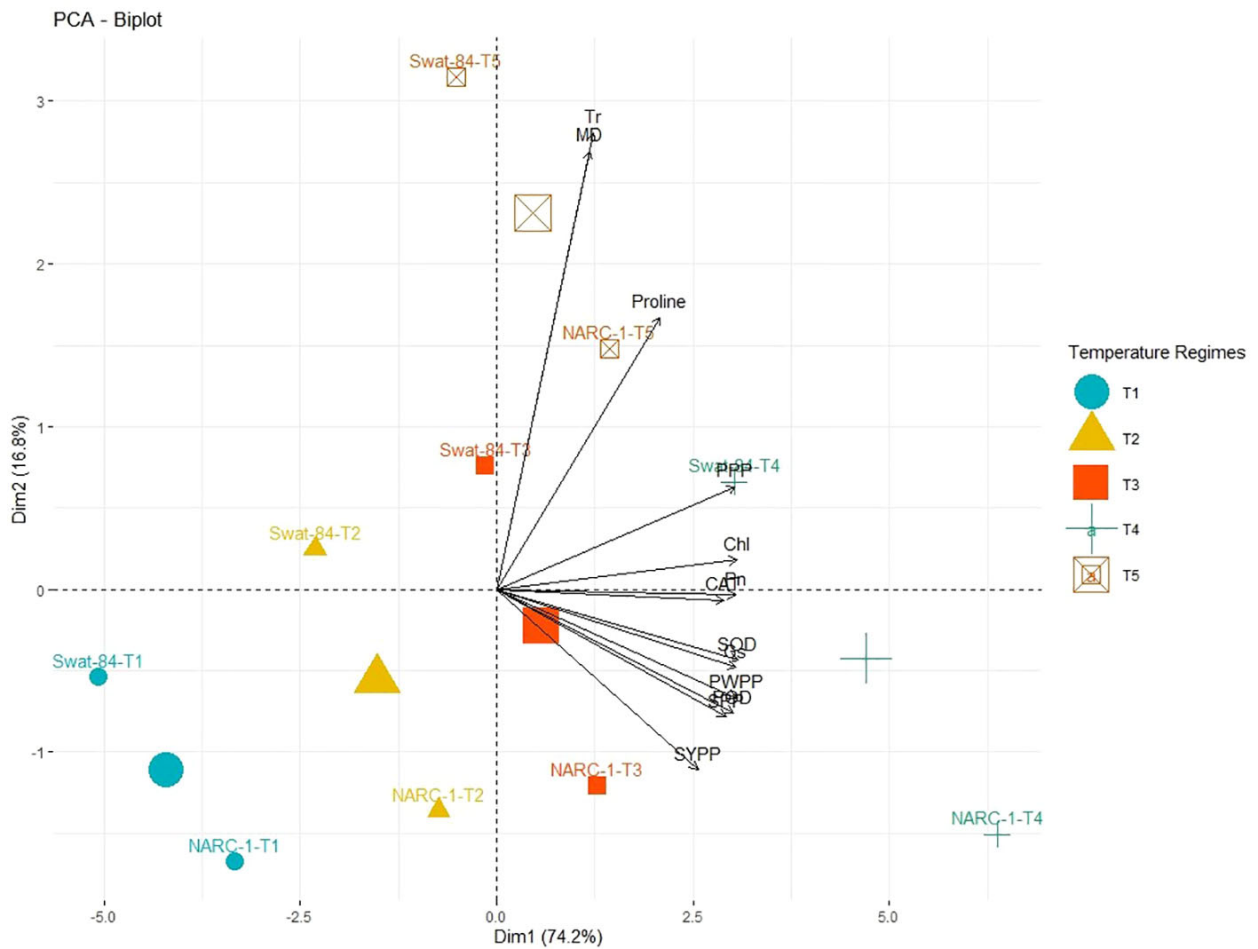
Principal component analysis (PCA) biplot of physiological, biochemical, and agronomic parameters grouped concerning their similarity and dissimilarity at varying regimes of day and night temperature. The varying lengths of vectors for origin indicate differential association with treatments, while the closeness of vectors indicates their strong association. The varying orientations of temperature regimes on biplots indicate that each treatment exhibits a different impact on trait association and expression. Chl, chlorophyll; CAT, catalase; Gs, stomatal conductance; MD, membrane damage; Pn, photosynthesis; POD, peroxidase; PPP, pods per plant; PWPP, pods weight per plant; SOD, superoxide dismutase; SPP, seeds per plant; SYPP, seed yield per plant; Tr, transpiration rate. T1 = 20°C/12°C, T2 = 25°C/17°C, T3 = 30°C/22°C, T4 = 35°C/27°C, and T5 = 40°C/32°C.

**Figure 4:**
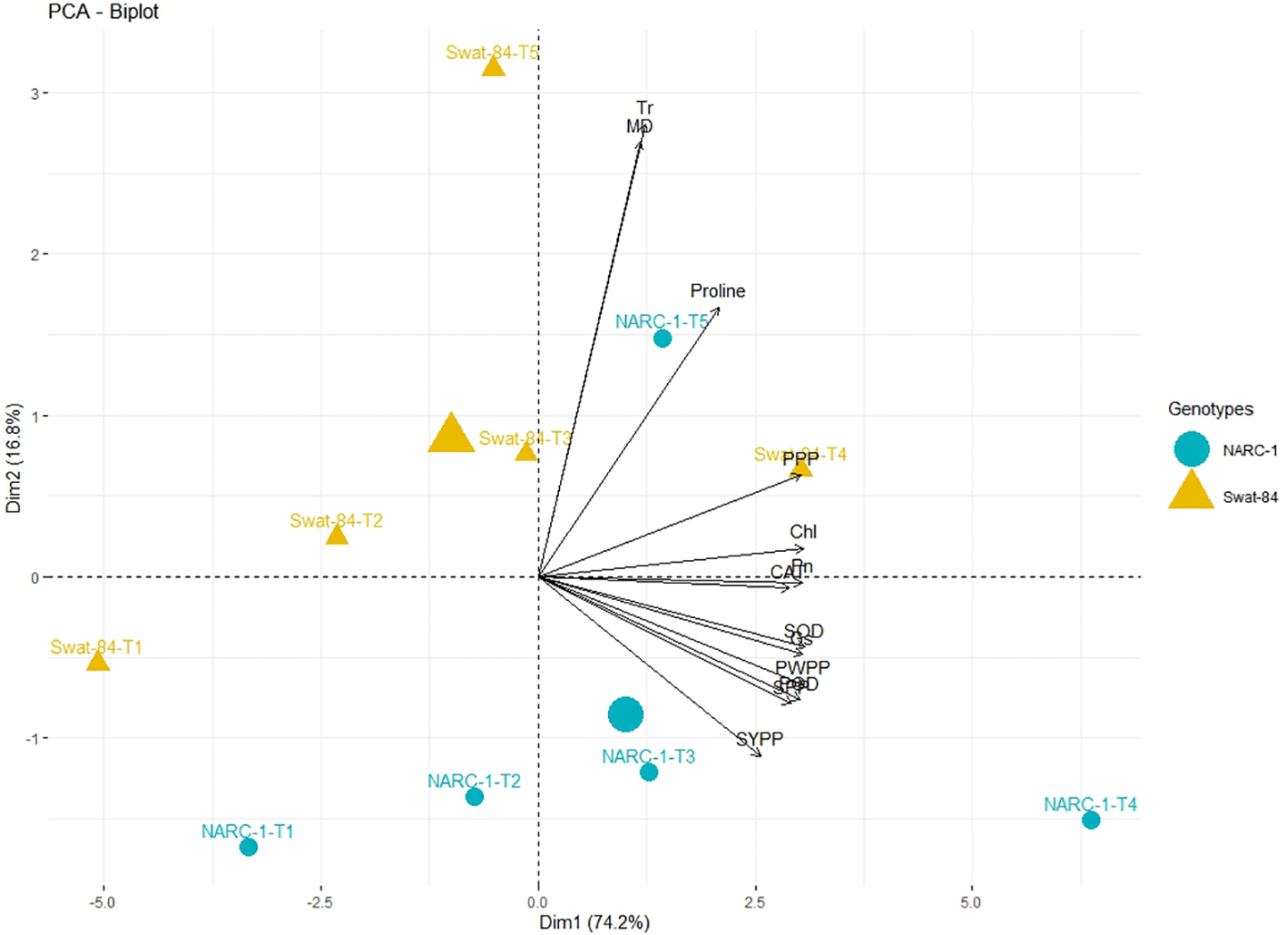
Principal component analysis (PCA) biplot of physiological, biochemical, and agronomic parameters grouped with respect to soybean cultivars based upon their similarity and dissimilarity. The varying lengths of vectors for origin indicate differential association with treatments, while the closeness of vectors indicates their strong association. The varying orientations of the cultivars on biplots indicate that each genotype exhibits a different impact on trait association and expression. Chl, chlorophyll; CAT, catalase; Gs, stomatal conductance; MD, membrane damage; Pn, photosynthesis; POD, peroxidase; PPP, pods per plant; PWPP, pods weight per plant; SOD, superoxide dismutase; SPP, seeds per plant; SYPP, seed yield per plant; Tr, transpiration rate.

**Figure 5:**
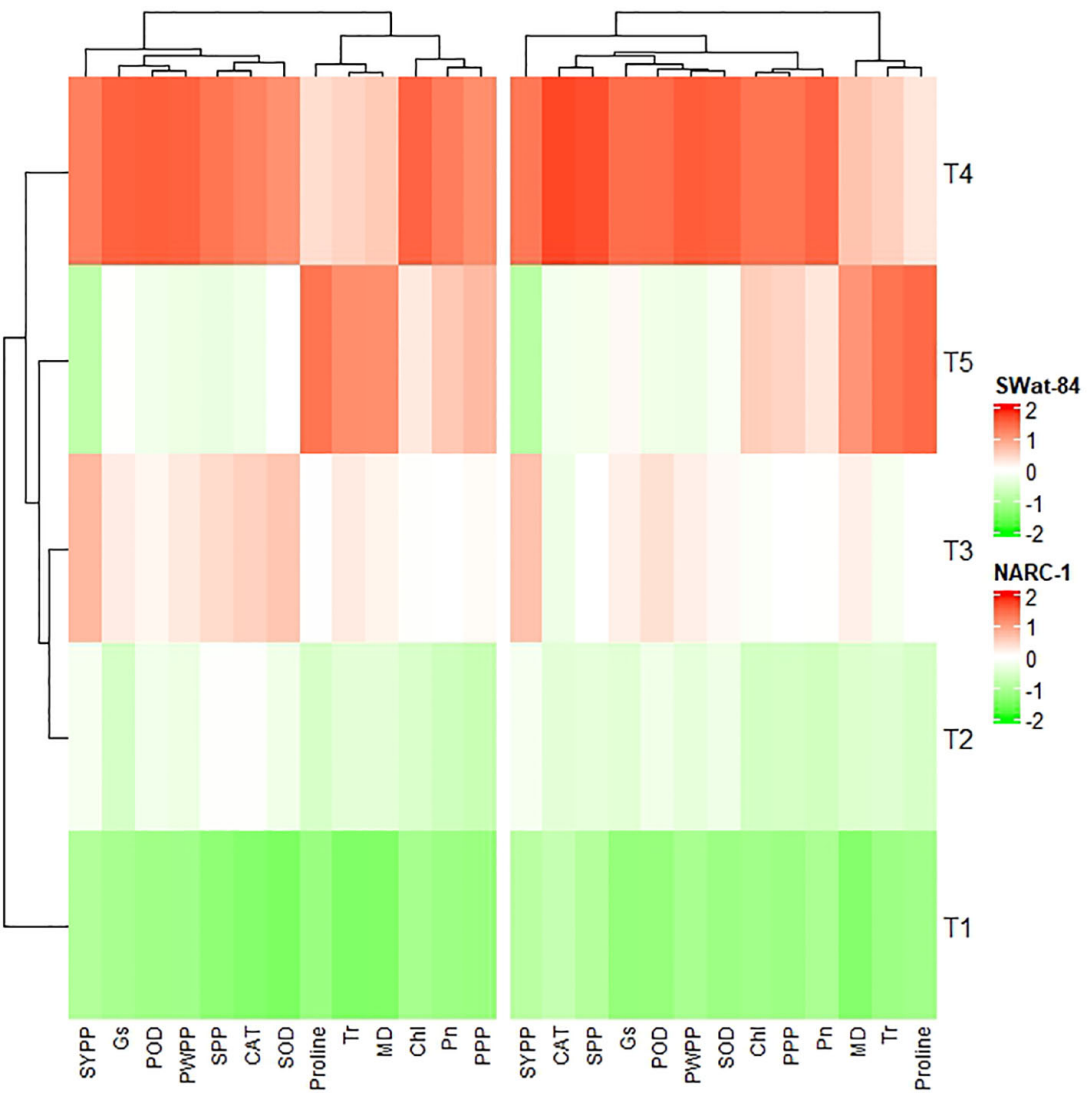
Heatmap cluster analysis depicting the differential impacts of varying regimes of day and night temperatures on the extent of expression of physiological, biochemical, and agronomic traits in two different soybean cultivars, Swat-84 (left) and NARC-1 (right). Chl, chlorophyll; CAT, catalase; Gs, stomatal conductance; MD, membrane damage; Pn, photosynthesis; POD, peroxidase; PPP, pods per plant; PWPP, pods weight per plant; SOD, superoxide dismutase; SPP, seeds per plant; SYPP, seed yield per plant; Tr, transpiration rate. T1 = 20°C/12°C, T2 = 25°C/17°C, T3 = 30°C/22°C, T4 = 35°C/27°C, and T5 = 40°C/32°C.

All traits showed a significant degree of paired association in a positive direction according to correlation analysis, with correlation coefficients ranging from 0.196 to 0.978 (**Figure 2**). Among physiological traits, Chl illustrated significantly high paired association with Pn, Gs, SOD, CAT, POD, PPP, PWPP, SPP, and SYPP excluding Tr, MD, and pro-line. In contrast, Tr, MD, and proline showed weak and non-significant associations with all biochemical, physiological, and agronomic traits (**Figure 2**). Additionally, except for Tr, MD, and proline, the activities of antioxidant enzymes (SOD, POD, and CAT) demonstrated a significant paired association with Chl, Pn, Gs, PWPP, SPP, and SYPP (**Figure 2**). Furthermore, all agronomic traits such as PPP, PWPP, SPP, and SYPP depicted strong correlations among themselves. Additionally, PCA showed varying dispersion of physiochemical and agronomic traits from biplot origin due to changing regimes of temperature that explicated changing expression and association pattern of traits due to variation of temperature (**Figure 3**). Furthermore, both genotypes showed a comparative difference in the orientation of trait clusters in the PCA graph under corresponding regimes of temperature that indicated the differential physiochemical responses of each genotype (**Figure 4**). Different cultivars of soybeans responded differently to applied temperature regimes, as demonstrated by PCA. In terms of the paired association of traits at T1, T2, T3, and T5, the genotype Swat-84 of the thermosensitive soybean showed a negative deviation (**Figures 3**, **4**). At the temperature T4, Swat-84 demonstrated a greater positive influence on the paired association of trait variables. The high dispersion of Swat-84 from the biplot origin further supports its high-rated sensitivity against varying temperature regimes (**Figures 3**, **4**). Conversely, at T1, the thermotolerant cultivar NARC-1 solely displayed a negative deviation concerning its origin, whereas, in the remaining temperature regimes, it demonstrated a positive influence on the trait-variable association (**Figures 3**, **4**). Additionally, at T4, NARC-1 had a significant effect on paired association. Overall, the close distribution of NARC-1 to the biplot origin at varying temperature regimes confirmed its tolerance against varying temperature regimes (**Figures 3**, **4**). Additionally, heatmap analysis further validated the results from PCA by grouping the traits into various clusters because of variations in the strength of their association with genotypes as well as with different day and night temperature regimes (**Figure 5**).

### 3.5 Relative expression analysis

**Figure 6:**
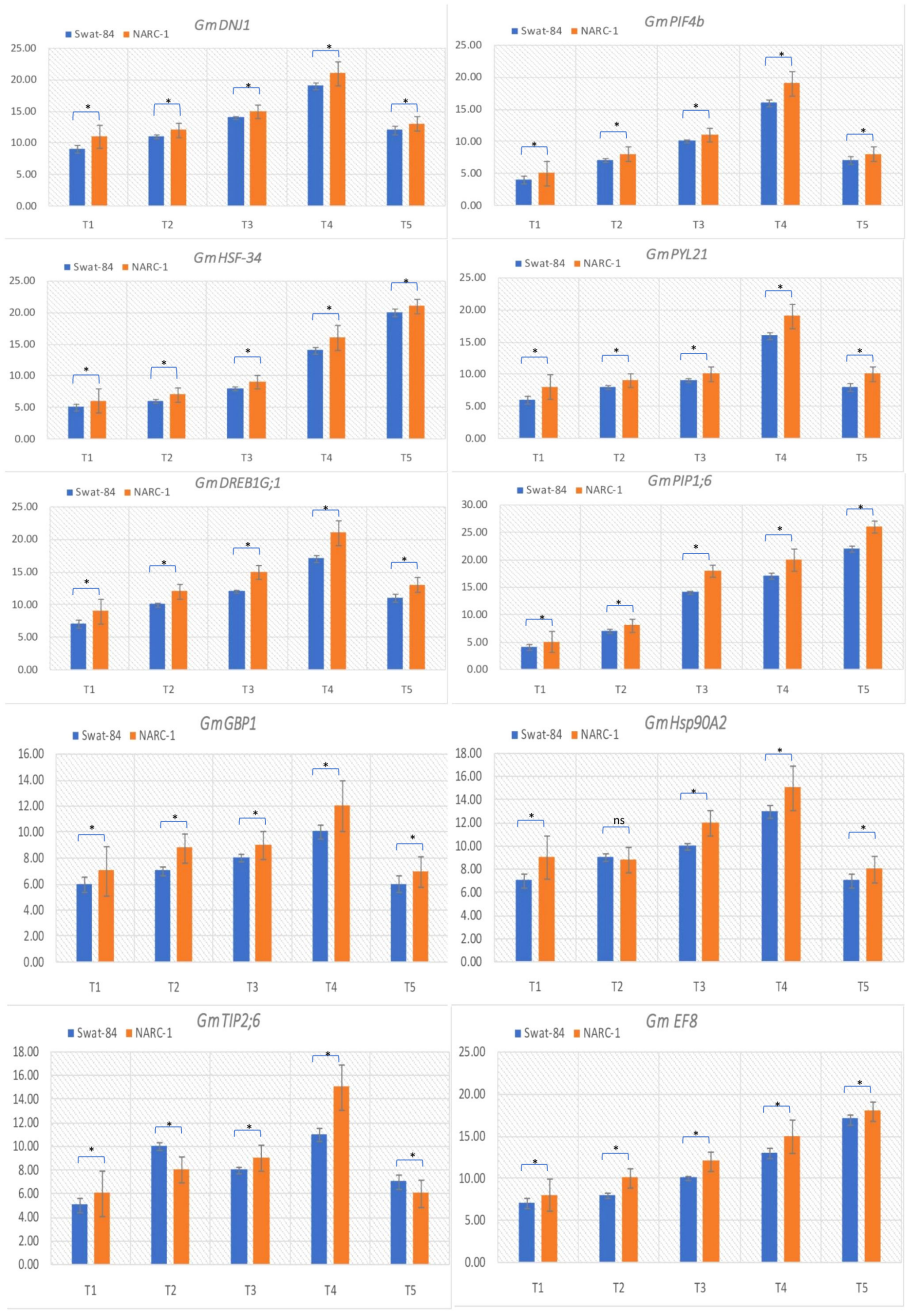
Relative expression analysis of heat stress-associated genes in soybean cultivars Swat-84 and NARC-1 under varying regimes of day and night temperatures. T1 = 20°C/12°C, T2 = 25°C/17°C, T3 = 30°C/22°C, T4 = 35°C/27°C, and T5 = 40°C/32°C. Bars show standard deviation ( ± SD). * indicates significant difference at p ≤ 0.01 and ns indicates non-significant difference.

**Figure 7:**
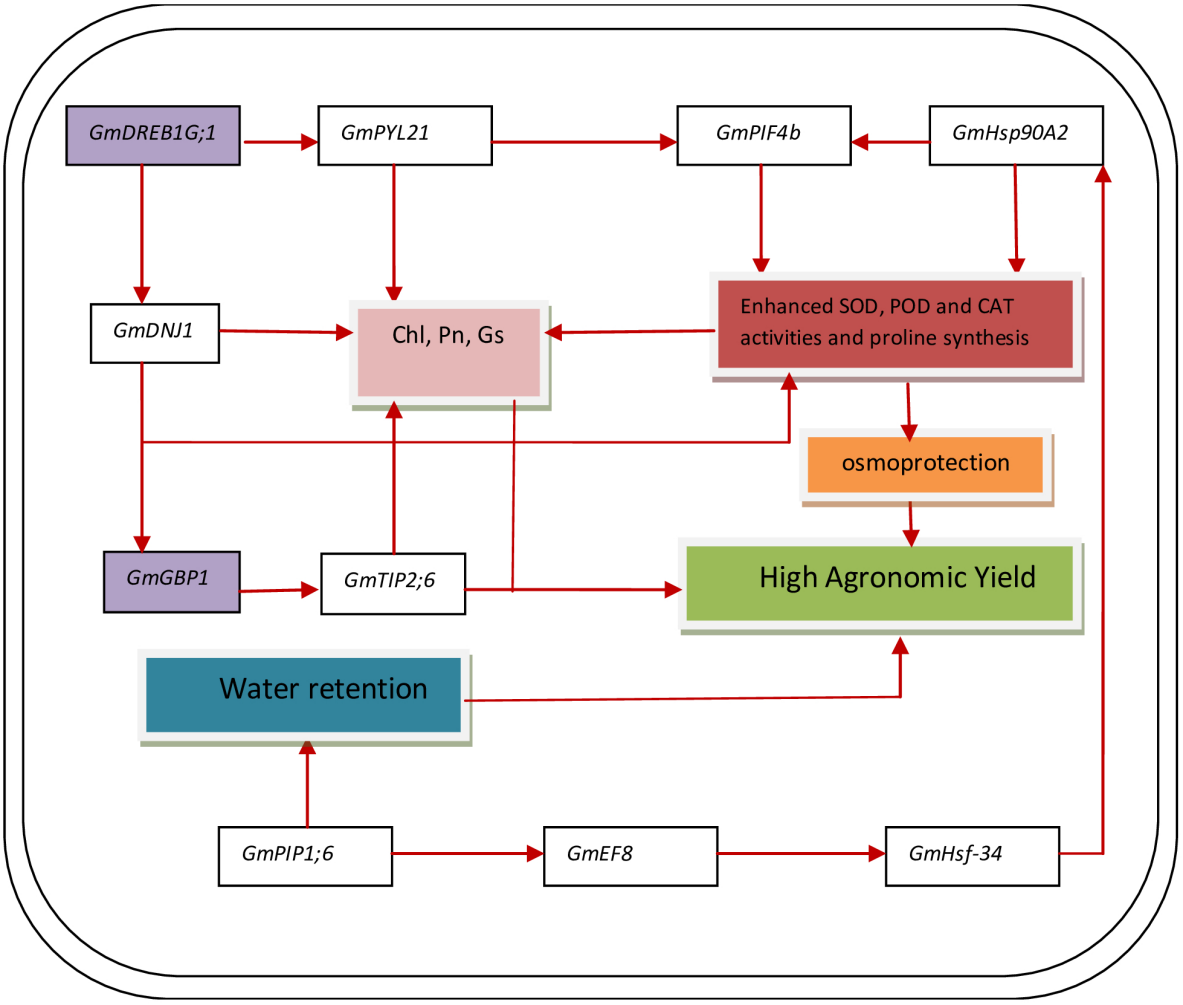
A general model representing how genes (*GmDNJ1*, *GmHSF-34*, *GmPYL21*, *GmPIF4b*, *GmPIP1;6*, *GmHsp90A2*, *GmTIP2;6*, and *GmEF8*) and TFs (*GmDREB1G;1* and *GmGBP1)* interact in both soybean genotypes for enhancing the physiochemical processes and increasing the agronomic yield when temperature regime is optimum. Chl, chlorophyll; CAT, catalase; Gs, stomatal conductance; Pn, photosynthesis; POD, peroxidase; SOD, superoxide dismutase. The red arrows indicate the activation of gene expression or process.

The relative expression of genes *GmDNJ1*, *GmDREB1G;1*, *GmHSF-34*, *GmPLY21*, *GmPIF4b*, *GmPIP1;6*, *GmGBP1*, *GmHsp90A2*, *GmTIP2;6*, and *GmEF8* showed significant (p ≤ 0.05) change with changing regimes of day and night temperatures (**Figure 6**). The transcript levels of *GmDNJ1*, *GmDREB1G;1GmPLY21*, and *GmPIF4b* significantly increased from T1 (20°C/12°C) to T4 (35°C/27°C) and dropped dramatically at T5 (40°C/32°C) in both soybean cultivars (**Figure 6**). The relative expression of growth-mediating gene *GmGBP1* increased significantly (p ≤ 0.05) from T1 (20°C/12°C) to T4 (35°C/27°C) and declined suddenly at T5 (40°C/32°C) in both soybean cultivars Swat-84 and NARC-1 (**Figure 6**). Correspondingly, *GmHsp90A2* recorded significantly (p ≤ 0.05) increasing relative expression from T1 (20°C/12°C) to T4 (35°C/27°C), which decreased dramatically at T5 (40°C/32°C) in both soybean cultivars (**Figure 6**). The gene *GmTIP2;6* illustrated significantly (p ≤ 0.05) high upregulation at T2 (25°C/17°C) and T4 (35°C/27°C) and low expression at T1 (20°C/12°C), T3 (30°C/22°C), and T5 (40°C/32°C) in both soybean cultivars (**Figure 6**). Conversely, the relative expression of genes *GmHSF-34*, *GmPIP1;6*, and *GmEF8* increased significantly (p ≤ 0.05) with increasing regimes of day and night temperatures from T1 (20°C/12°C) to T5 (40°C/32°C) in both soybean cultivars (**Figure 6**). Overall, the relative expression of all genes was higher in NARC-1 as compared to Swat-84 at all corresponding regimes of temperatures (**Figure 6**). Overall, the overexpression of all genes was consistent with increased activities of antioxidant enzymes (SOD, POD, and CAT), proline content, and physiological traits (Chl, Gs, and Pn) as shown in **Figure 7**.

## 4 Discussion

The current study was performed to assess the impact of varying regimes of day and night temperatures on the physiochemical, genetic, and agronomic traits of soybean cultivars. Like any other living organism, plants are equipped with the tendency to respond to their metrological environment (Raza et al., 2019). In this context, temperature is an eminent environmental factor regulating plant physiochemical processes both directly and indirectly (Osei et al., 2023). The optimum temperature is a prerequisite to regulate the plant’s essential physiological processes including chlorophyll biosynthesis, Pn, Gs, and Tr (Muhammad et al., 2021). Photosynthetic machinery in leaves is a tentatively logical place to begin particularly when speculating the effects of temperature on crop photosynthesis, as various steps of photosynthesis are highly temperature-dependent (Moore et al., 2021). From a biochemical perspective, CO_2_ assimilation is determined by the activation and efficiency of the enzyme Rubisco at ambient temperature (Muhammad et al., 2021). Additionally, enhanced enzymatic activity till optimum temperature triggers the function of the photosystem due to an increase in chlorophyll biosynthesis (Moore et al., 2021). However, temperatures beyond the optimum impede the function of the photosystem and hamper the Rubisco activation in addition to the decrease in chlorophyll content, which collectively result in a substantial reduction in Pn and CO_2_ assimilation in soybean as reviewed by Herritt and Fritschi (2020). Consistent with these findings, the current study reported a significant decline in chlorophyll content and Pn below and beyond the ambient regime of the temperature T4 (35°C/27°C) (**Figure 1**). In contrast, stomatal behavior is significantly important to control the gaseous exchange between plant interior and atmosphere (Driesen et al., 2020). Moreover, Gs is imperative for CO_2_ uptake and leaf water Tr in addition to an indicator of increased Pn (Xu et al., 2016). The tendency of plants to sustain Gs, Tr, and Pn under wide ranges of temperature is directly correlated with their potential to tolerate wide regimes of temperatures (Driesen et al., 2020). The decline in chlorophyll pigment below or beyond the ambient temperature is respectively a consequence of the decline in Chl synthase activity and peroxidation of chloroplast and thylakoid membrane (Hasanuzzaman et al., 2013). Furthermore, Alsajri et al. (2019) and Djanaguiraman et al. (2019) respectively evaluated the effect of specific and varying regimes of day and night temperatures on physiological and agronomic traits of soybean and found a strong relation between chlorophyll, Gs, Pn, and yield-related traits. The Gs is a measure of stomata opening, which determines the uptake of CO_2_ and release of water vapors (Farquhar and Sharkey, 1982). Interestingly the stomatal Gs was maximum at T4 (35°C/27°C), while Tr was maximum at T5 (40°C/32°C) (**Figure 1**). This was probably due to the robust CO_2_ fixation at T4 (35°C/27°C) as confirmed by the maximum rate of Pn at this temperature. Furthermore, at T4 (35°C/27°C), the CO_2_ fixation contributed maximally to Gs as compared to Tr. Moreover, temperature above optimum results in decreased membrane stability and enhanced Tr (Jianing et al., 2022). This reflects that all these processes strongly adhere to and are affected by the temperature as a unit as indicated in **Figures 2**, **3**. In contrast, a consistent increase in temperature enhances the level of ROS in plant cellular systems, inducing lipid peroxidation in cell membranes that disrupts the structural integrity of the membranes (Awasthi et al., 2015). An increase in membrane damage leads to the inhibition of plant vital physiological processes including Pn. However, plants are not passive entities; instead, they respond actively to metrological factors intended to perturb plant homeostasis. In this way, the increasing regimes of temperature trigger the activities of antioxidant enzymes that are actively involved in cellular homeostasis and ROS scavenging (Rajput et al., 2021). Siebers et al. (2015) noticed that the activities of CAT, SOD, and POX increase in soybean due to oxidative stress caused by high temperatures. Consistent with these findings, the current study reported a dynamic increase in the activities of antioxidant enzymes with increasing regimes of temperatures from T1 (20°C/12°C) to T4 (35°C/27°C) to mask the effect of ROS through scavenging (**Figure 1**). Correspondingly, proline acts as an excellent osmolyte in plants subjected to various types of stresses by playing its role as an antioxidative defense and signaling molecule (Hayat et al., 2012). Consistently increasing variations of temperature facilitate the speedy production of proline, which not only balances cellular water and osmotic potential but also activates the physiological and biochemical processes monitoring plant yield directly and indirectly as confirmed by the present study (**Figure 1**). Temporal and specific variations in temperature over soybean growing areas affect soybean yield (Alsajri et al., 2022). Plants continuously struggle for survival under varying regimes of temperature. Plant endures, to some extent, the dynamically changing temperature in various ways, specifically by producing osmo-protectants with a tendency to modify the antioxidant system for reestablishing the cell redox ionic homeostasis (Ghosh et al., 2021). In this perspective, it is important to consider the impact of temperature while optimizing the metrological conditions for the cultivation of soybeans (Yang et al., 2023). The temperature has a direct relationship with physiological and biochemical processes determining agronomic productivity (Vogel et al., 2021). Additionally, the variation of temperature exhibits multifarious, and often detrimental, changes in plant physiological processes, development, metabolism, and agronomic yield (Vogel et al., 2021; Yang et al., 2023). This strong connection was further confirmed through correlation and PCA (**Figures 2**–**4**). The optimal regime for soybeans is essentially important from seedling to grain filling for efficient physiological processes to maximize the agronomic yield in terms of PPP, PWPP, SPP, and SYPP (Alsajri et al., 2022; Jianing et al., 2022). The agronomic yield varies corresponding to physiological yield at changing regimes of day and night temperatures. Consistent with these studies, the current study reported a complete parallelism between the variation in physiological and biochemical traits below and beyond the optimum temperature T4 (35°C/27°C) (**Figures 1**–**3**), which was in accordance with the results of Alsajri et al. (2019); Yang et al. (2023), and Choi et al. (2016). Additionally, the paired association of physiological, biochemical, and agronomic traits varies not only with changing regimes of temperatures but also with the nature of the cultivar used. This speculation was further confirmed by PCA and heatmap studies (**Figures 4**, **5**). To unravel the complex response of soybean genotypes to changing regimes of temperature, both cultivars Swat-84 and NARC-1 were further evaluated for their genetic response. In the present study, the expression of temperature-related genes varied significantly in both soybean cultivars due to varying regimes of day and night temperatures (**Figure 6**). The *GmDNJ1*, a type 1 HSP-40 protein, has a tendency to sustain overall soybean growth under heat stress by modulating the activities of various enzymes involved in ROS scavenging and chlorophyll synthesis in addition to inhibition of protein catabolism (Li et al., 2021). Correspondingly, the current study recorded the maximum expression of *GmDNJ1* at T4 (35°C/27°C), the temperature at which the activity of antioxidant enzymes and amount of chlorophyll, Pn, and Gs were maximum (**Figures 1**, **6**). Furthermore, the upregulation of DREB1 target genes such as *GmDREB1B;1*, *GmDREB1C;1*, and *GmDREB1F;1* play an essential role in triggering the expression of various genes under heat stress (Kidokoro et al., 2015). The enhanced expression of *GmDREB1G;1* activates the expression of other heat-responsive genes such as *GmPYL21* that ensure plant normal physiological and molecular processes (Kidokoro et al., 2015). This was the most probable reason for the maximum transcript level of *GmDREB1G;1* and *GmPYL21* at T4 (35°C/27°C) in both soybean cultivars in proportion to enhanced physiochemical activities (**Figures 1**, **6**). Heat shock transcription factors (HSFs) play a significant role in responses against heat stress. Additionally, HSFs in association with heat stress elements activate HSPs in plants to strengthen their thermotolerance (Li et al., 2014). Moreover, Guo et al. (2016) found that overexpression of *GmHSF1A* strengthens the thermotolerance of transgenic soybeans due to activation of *GmHSP70*, *GmHSP22*, and *GmHSP90A2* under heat stress. Correspondingly, the activity of *GmHSF-34* increased consistently till T5 (40°C/32°C) in both soybean cultivars, and plants retained their activities essential for survival (**Figure 6**). Furthermore, Arya et al. (2023) confirmed through qRT-PCR analysis that the soybean cultivars with upregulating *GmPIF4b* had higher heat shock proteins *GmHSP90A2* and transcripts of heat shock factor. Hence, the gene *GmPIF4b* regulates multiple morphological (plant height, number of branches, and leaf surface area) and physiological traits (chlorophyll and proline) for better crop yield under high temperatures. Accordingly, the present study recorded a complementary increase in the expression of the aforementioned genes at T4 (35°C/27°C) in addition to high proline and chlorophyll contents (**Figures 1**, **6**). The overexpressing soybean plasma membrane intrinsic protein 1;6 (*GmPIP1;6*) is an aquaporin with multiple functions that regulate plant normal water uptake, photosynthesis, and grain filling under saline stress as confirmed by Zhou et al. (2014). The current study reported similar results under temperature regime T4 (35°C/27°C) in addition to enhanced Pn, Gs, and chlorophyll (**Figures 1**, **6**). The overexpression of soybean gene *GmHsp90A2* under high temperatures is associated with countering oxidative stress and maximizing chlorophyll production as reported by Huang et al. (2019). Correspondingly, the current study recorded the maximum expression of *GmHsp90A2* at T4 (35°C/27°C), the temperature at which chlorophyll production and antioxidant activities were also maximum (**Figures 1**, **6**). Similarly, Zhao et al. (2013) and Feng et al. (2019) respectively found that high expression of *GmGBP1* and *GmTIP2;6* produces specific proteins that modulate agronomic growth under specific temperatures. In agreement with these findings, the current study recorded high expressions of *GmGBP1* and *GmTIP2;6* in both soybean cultivars at temperature regime T4 (35°C/27°C), where the highest agronomic yield was recorded (**Figures 1**, **6**). Unlike *GmDNJ1*, *GmDREB1G;1*, *GmPYL21*, *GmPIF4b*, *GmHsp90A2*, *GmGBP1*, and *GmTIP2;6*, the genes *GmHSF-34*, *GmPIP1;6*, and *GmEF8* showed the highest level of transcript at T5 (40°C/32°C) in both soybean cultivars, which was consistent with the rise in proline content (**Figures 1**, **6**). In fact, *GmEF8*, *GmHSF-34*, and *GmPIP1;6* respectively enhance proline, HSP, and water uptake when soybean faces temperature stress, and they have a protective role *via* osmolyte adjustments (Zhou et al., 2014; Zhang et al., 2022; Arya et al., 2023). High-temperature regimes accelerate the loss of water from plant surfaces due to transpiration and create a water-deficit environment within plants (Fahad et al., 2017). Plants, being active entities, respond to water-deficit conditions through osmotic adjustments. In response to high temperatures, plants alter their metabolism in different ways, particularly by enhancing the production of solutes that re-establish cellular homeostasis and redox balance by organizing proteins, modifying the antioxidant system, and maintaining cell turgor owing to osmotic adjustments (Hasanuzzaman et al., 2013). The plant copes with heat stress through the synthesis of compatible solutes known as osmoprotectants that regulate the water contents. Among extensively studied osmolytes, proline is one of the most effective compatible solutes and is ranked as the top osmoprotectant in plants (Siddique et al., 2018). Despite proline being an important signaling molecule, the most important functions of accumulated proline are osmoregulation, cell membrane maintenance, and protein stability under water-deficit conditions (Liang et al., 2013). The present study confirmed these essential roles of proline under increasing temperature regimes that were consistent with the expression of proline-regulating genes (**Figures 1**, **6**). Additionally, the regulation of aquaporins (AQPs) is vital for maintaining water balance during heat stress, as excessive transpiration can lead to water loss and dehydration (Afzal et al., 2016). Aquaporins are channel proteins facilitating the across-membrane transport of water, which plays an important role in biological processes (Li et al., 2019). In this perspective, the overexpression *GmPIP1;6* retains the water conductance to enhance the thermotolerance during heat stress, which is not an astonishing phenomenon. Moreover, *GmEF8* has tendency to regulate the production of proline through interaction with other genes involved in the proline synthesis pathway (Kishor et al., 2005). Although high temperature increases the level of HSP in plants, to some extent, it is correlated with the expression of antioxidant enzymes as reported by Wang et al. (2021) in rice. Perhaps, the decreased activities of antioxidant enzymes at T5 (40°C/32°C) were the cause of the reduced expression of *GmHsp90A2*. Like all other organisms, plant processes including physiological and biochemical are genetically regulated. The change in the transcript level of genes with different temperature regimes was complementary to the variation of physiological and biochemical traits that determine the crop agronomic productivity (**Figure 7**). In general, the soybean cultivar NARC-1 showed significantly high values of physiological, biochemical, agronomic, and genetic traits as compared to Swat-84 at corresponding regimes of temperatures, which confirms that NARC-1 is more tolerant against temperature variations as compared to Swat-84. Overall, the current study proved that physiological, biochemical, agronomic, and genetic traits are deeply linked and susceptible to varying regimes of temperature. Different regimes impact the physiological and agronomic productivity differently; however, thorough investigation through genetic study proved that the optimum day and night temperature for soybean from the vegetative stage to the grain-filling stage was T4 (35°C/27°C), whose slight variation impacts its productivity significantly. Soybean is a highly temperature-sensitive crop; therefore, breeding against temperature stress should be an integral part of soybean future breeding programs. To devise an efficient breeding program to breed soybeans with tolerance against temperature stress, there is a need to consolidate the screening techniques of genotypes. Temperature stress is a major problem in arid regions where farmers face substantial losses in soybean production due to inappropriate selection of genotypes that are not adaptable to the metrological conditions of that particular area. Therefore, soybean germplasm requires comprehensive screening at molecular, biochemical, physiological, and morphological levels to optimize their adaptability in regions where temperature variation is a potential constraint at the terminal stages of soybean.

## Data availability statement

The original contributions presented in the study are included in the article/supplementary material. Further inquiries can be directed to the corresponding author.

## Author contributions

CD: Methodology, Data Curation, Writing – original draft. FA: Methodology, Data Curation, Writing – review & editing. MR: Formal analysis, Writing – original draft. TZ: Methodology, Data Curation, Writing – original draft. MMJ: Formal analysis, writing – original draft. RA: Formal analysis, Writing – original draft. A-HG: Writing – review & editing. AA-D: Methodology, Supervision, Writing – review & editing. TK: Methodology, Data Curation, Writing – review & editing. SHY: Supervision, Writing – review & editing. ZS: Supervision, Writing – review & editing.

## References

[B1] Afzal Z. Howton T. C. Sun Y. Mukhtar M. S. (2016). The roles of aquaporins in plant stress responses. J. Dev. Biol. 4 (1), 9. doi: 10.3390/jdb4010009 29615577 PMC5831814

[B2] Alsajri F. A. Singh B. Wijewardana C. Irby J. T. Gao W. Reddy K. R. (2019). Evaluating soybean cultivars for low- and high-temperature tolerance during the seedling growth stage. Agronomy 9 (1), 13. doi: 10.3390/agronomy9010013

[B3] Alsajri F. A. Wijewardana C. Bheemanahalli R. Irby J. T. Krutz J. Golden B.. (2022). Morpho-physiological, yield, and transgenerational seed germination responses of soybean to temperature. Front. Plant Sci. 13, 839270. doi: 10.3389/fpls.2022.839270 35392514 PMC8981302

[B4] Alsajri F. A. Wijewardana C. Rosselot R. Singh B. Krutz L. J. Gao W.. (2020). Temperature effects on soybean seedling shoot and root growth and developmental dynamics. J. Miss. Acad. Sci. 65 (3).

[B5] Arya H. Singh M. B. Bhalla P. L. (2023). Overexpression of GmPIF4b affects morpho-physiological traits to reduce heat-induced grain loss in soybean. Plant Physiol. Biochem. 206, 108233. 38134737 10.1016/j.plaphy.2023.108233

[B6] Asad S. A. Wahid M. A. Farina S. Ali R. Muhammad F. (2020). Soybean production in Pakistan: experiences, challenges and prospects. IJAB 24 (4), 995–1005.

[B7] Awasthi R. Bhandari K. Nayyar H. (2015). Temperature stress and redox homeostasis in agricultural crops. Front. Environ. Sci. 3, 11. doi: 10.3389/fenvs.2015.00011

[B8] Bienias A. Góralska M. Masojć P. Milczarski P. Myśków B. (2020). The GAMYB gene in rye: sequence, polymorphisms, map location, allele-specific markers, and relationship with α-amylase activity. BMC Genomics 21, 1–15. doi: 10.1186/s12864-020-06991-3 PMC7444254 32831010

[B9] Choi D. H. Ban H. Y. Seo B. S. Lee K. J. Lee B. W. (2016). Phenology and seed yield performance of determinate soybean cultivars grown at elevated temperatures in a temperate region. PloS One 11 (11), e0165977. doi: 10.1371/journal.pone.0165977 27812185 PMC5094742

[B10] Di F. Jian H. Wang T. Chen X. Ding Y. Du H.. (2018). Genome-wide analysis of the PYL gene family and identification of PYL genes that respond to abiotic stress in Brassica napus. Genes 9 (3), 156. doi: 10.3390/genes9030156 29534558 PMC5867877

[B11] Ding X. Guo Q. Li Q. Gai J. Yang S. (2020). Comparative transcriptomics analysis and functional study reveal important role of high-temperature stress response gene GmHSFA2 during flower bud development of CMS-based F1 in soybean. Front. Plant Sci. 11, 600217. doi: 10.3389/fpls.2020.600217 33384706 PMC7770188

[B12] Djanaguiraman M. Boyle D. L. Welti R. Jagadish S. V. K. Prasad P. V. V. (2018). Decreased photosynthetic rate under high temperature in wheat is due to lipid desaturation, oxidation, acylation, and damage of organelles. BMC Plant Biol. 8 (1), 55. doi: 10.1186/s12870-018-1263-z PMC5887265 29621997

[B13] Djanaguiraman M. Schapaugh W. Fritschi F. Nguyen H. Prasad P. V. (2019). Reproductive success of soybean (*Glycine max* L. Merril) cultivars and exotic lines under high daytime temperature. Plant Cell Environ. 42 (1), 321–336. 30095867 10.1111/pce.13421

[B14] Driesen E. Van den Ende W. De Proft M. Saeys W. (2020). Influence of environmental factors light, CO_2_, temperature, and relative humidity on stomatal opening and development: a review. Agronomy 10, 1975. doi: 10.3390/agronomy10121975

[B15] Fahad S. Bajwa A. A. Nazir U. Anjum S. A. Farooq A. Zohaib A.. (2017). Crop production under drought and heat stress: plant responses and management options. Front. Plant Sci., 1147. doi: 10.3389/fpls.2017.01147 28706531 PMC5489704

[B16] Farquhar G. D. Sharkey T. D. (1982). Stomatal conductance and photosynthesis. Ann. Rev. Plant Physiol. 33 (1), 317–345. doi: 10.1146/annurev.pp.33.060182.001533

[B17] Feng Z. J. Liu N. Zhang G. W. Niu F. G. Xu S. C. Gong Y. M. (2019). Investigation of the AQP family in soybean and the promoter activity of TIP2; 6 in heat stress and hormone responses. Int. J. Mol. Sci. 20, 262. doi: 10.3390/ijms20020262 30634702 PMC6359280

[B18] Ghosh U. K. Islam M. N. Siddiqui M. N. Khan M. A. R. (2021). Understanding the roles of osmolytes for acclimatizing plants to changing environment: a review of potential mechanism. PlantSignal. Behav. 16 (8), 1913306. doi: 10.1080/15592324.2021.1913306 PMC8244753 34134596

[B19] Guo M. Liu J. H. Ma X. Luo D. X. Gong Z. H. Lu M. H. (2016). The plant heat stress transcription factors (HSFs): structure, regulation, and function in response to abiotic stresses. Front. Plant Sci. 7, 114. doi: 10.3389/fpls.2016.00114 26904076 PMC4746267

[B20] Hasanuzzaman M. Nahar K. Alam M. M. Roychowdhury R. Fujita M. (2013). Physiological, biochemical, and molecular mechanisms of heat stress tolerance in plants. Int. J. Mol. Sci. 14, 9643–9684. doi: 10.3390/ijms14059643 23644891 PMC3676804

[B21] Hayat S. Hayat Q. AlYemeni M. N. Wani A. S. Pichtel J. Ahmad A. (2012). Role of proline under changing environments: a review. Plant Signal. Behav. 7 (11), 1456–1466. doi: 10.4161/psb.21949 22951402 PMC3548871

[B22] Herritt M. T. Fritschi F. B. (2020). Characterization of photosynthetic phenotypes and chloroplast ultrastructural changes of soybean (*Glycine max*) in response to elevated air temperatures. Front. Plant Sci. 11, 153. doi: 10.3389/fpls.2020.00153 32210985 PMC7069378

[B23] Hsieh E. J. Cheng M. C. Lin T. P. (2013). Functional characterization of an abiotic stress-inducible transcription factor AtERF53 in Arabidopsis thaliana. Plant Mol. Biol. 82, 223–237. doi: 10.1007/s11103-013-0054-z 23625358

[B24] Huang Y. Xuan H. Yang C. Guo N. Wang H. Zhao J.. (2019). *GmHsp90A2* is involved in soybean heat stress as a positive regulator. Plant Sci. 285, 26–33. doi: 10.1016/j.plantsci.2019.04.016 31203891

[B25] Jianing G. Yuhong G. Yijun G. Rasheed A. Qian Z. Zhiming X.. (2022). Improvement of heat stress tolerance in soybean (*Glycine max* L), by using conventional and molecular tools. Front. Plant Sci. 13 993189. doi: 10.3389/fpls.2022.993189 PMC9549248 36226280

[B26] Kapilan R. Vaziri M. Zwiazek J. J. (2018). Regulation of aquaporins in plants under stress. Biol. Res. 51 (1), 4. doi: 10.1186/s40659-018-0152-0 29338771 PMC5769316

[B27] Kidokoro S. Watanabe K. Ohori T. Moriwaki T. Maruyama K. Mizoi J.. (2015). Soybean DREB1/CBF-type transcription factors function in heat and drought as well as cold stress-responsive gene expression. Plant J. 81 (3), 505–518. doi: 10.1111/tpj.12746 25495120

[B28] Kishor P. K. Sangam S. Amrutha R. N. Laxmi P. S. Naidu K. R. Rao K. S.. (2005). Regulation of proline biosynthesis, degradation, uptake and transport in higher plants: its implications in plant growth and abiotic stress tolerance. Curr. Sci. 10, 424–438.

[B29] Li K. P. Wong C. H. Cheng C. C. Cheng S. S. Li M. W. Mansveld S.. (2021). GmDNJ1, a type-I heat shock protein 40 (HSP40), is responsible for both Growth and heat tolerance in soybean. Plant Direct 5 (1), e00298. doi: 10.1002/pld3.298 33532690 PMC7833466

[B30] Li P. S. Yu T. F. He G. H. Chen M. Zhou Y. B. Chai S. C.. (2014). Genome-wide analysis of the Hsf family in soybean and functional identification of GmHsf-34 involvement in drought and heat stresses. BMC Genomics 15 (1), 1009. doi: 10.1186/1471-2164-15-1009 25416131 PMC4253008

[B31] Li S. Liu J. An Y. Cao Y. Liu Y. Zhang J.. (2019). MsPIP2; 2, a novel aquaporin gene from Medicago sativa, confers salt tolerance in transgenic Arabidopsis. Environ.Exp. Bot. 165, 39–52. doi: 10.1016/j.envexpbot.2019.05.020

[B32] Liang X. Zhang L. Natarajan S. K. Becker D. F. (2013). Proline mechanisms of stress survival. Antioxidants Redox Signaling 19 (9), 998–1011. doi: 10.1089/ars.2012.5074 23581681 PMC3763223

[B33] Mathur S. Agrawal D. Jajoo A. (2014). Photosynthesis: response to high temperature stress. J. Photochem. Photobiol. B. 137, 116–126. doi: 10.1016/j.jphotobiol.2014.01.010 24796250

[B34] McGraw-Hill C. (2008). Statistix 8.1 (Analytical software, Tallahassee, Florida) (Florida, USA: Maurice/Thomas text).

[B35] Moore C. E. Meacham-Hensold K. Lemonnier P. Slattery R. A. Benjamin C. Bernacchi C. J.. (2021). The effect of increasing temperature on crop photosynthesis: from enzymes to ecosystems. J. Exp. Bot. 72 (8), 2822–2844. doi: 10.1093/jxb/erab090 33619527 PMC8023210

[B36] Muhammad I. Shalmani A. Ali M. Yang Q. H. Ahmad H. Li F. B. (2021). Mechanisms regulating the dynamics of photosynthesis under abiotic stresses. Front. Plant Sci. 11, 615942. doi: 10.3389/fpls.2020.615942 33584756 PMC7876081

[B37] Ortiz A. C. De Smet I. Sozzani R. Locke A. M. (2022). Field-grown soybean shows genotypic variation in physiological and seed composition responses to heat stress during seed development. Environ. Exp. Bot. 195, 104768. doi: 10.1016/j.envexpbot.2021.104768

[B38] Osei E. Jafri S. H. Saleh A. Gassman P. W. Gallego O. (2023). Simulated climate change impacts on corn and soybean yields in Buchanan County, Iowa. Agriculture 13, 268. doi: 10.3390/agriculture13020268

[B39] Purcell L. C. Salmeron M. Ashlock L. (2014). Soybean growth and development. Arkans. Soyb. Product. Handbook MP. 197, 1–8.

[B40] Rajput V. D. Harish Singh R. K. Verma K. K. Sharma L. Quiroz-Figueroa F. R.. (2021). Recent developments in enzymatic antioxidant defence mechanism in plants with special reference to abiotic stress. Biology 10, 267. doi: 10.3390/biology10040267 33810535 PMC8066271

[B41] Raza A. Razzaq A. Mehmood S. S. Zou X. Zhang X. Lv Y.. (2019). Impact of climate change on crops adaptation and strategies to tackle its outcome: a review. Plants 8, 34. doi: 10.3390/plants8020034 30704089 PMC6409995

[B42] RStudio Team. (2020). RStudio: integrated development for R (PBC, Boston, MA: RStudio). Available at: http://www.rstudio.com/.

[B43] Sachdev S. Ansari S. A. Ansari M. I. Fujita M. Hasanuzzaman M. (2021). Abiotic stress and reactive oxygen species: generation, signaling, and defense mechanisms. Antioxidants 10, 277. doi: 10.3390/antiox10020277 33670123 PMC7916865

[B44] Sairam R. K. Deshmukh P. S. Shukla D. S. (1997). Tolerance to drought and temperature stress in relation to increased antioxidant enzyme activity in wheat. J. Agron. Crop Sci. 178, 171–177. doi: 10.1111/j.1439-037X.1997.tb00486.x

[B45] Sasi S. Venkatesh J. Daneshi R. F. Gururani M. A. (2018). Photosystem II extrinsic proteins and their putative role in abiotic stress tolerance in higher plants. . Plants 7, 100. doi: 10.3390/plants7040100 30441780 PMC6313935

[B46] Siddique A. Kandpal G. Kumar P. (2018). Proline accumulation and its defensive role under diverse stress condition in plants: an overview. J. Pure Appl. Microbiol. 12 (3), 1655–1659. doi: 10.22207/JPAM.12.3.73

[B47] Siebers M. H. Yendrek C. R. Drag D. Locke A. M. Rios Acosta L.. (2015). Heat waves imposed during early pod development in soybean (*Glycine max*) cause significant yield loss despite a rapid recovery from oxidative stress. Glob. Change Biol. 21 (8), 3114–3125. doi: 10.1111/gcb.12935 25845935

[B48] Tariq A. Jabeen Z. Farrakh S. Noreen K. Arshad W. Ahmed H.. (2022). Exploring the genetic potential of Pakistani soybean cultivars through RNA-seq based transcriptome analysis. Mol. Biol. Rep. 49, 2889–2897. doi: 10.1007/s11033-021-07104-3 35088376

[B49] Ul-Haq S. Khan A. Ali M. Khattak A. M. Gai W. X. Zhang H. X.. (2019). Heat shock proteins: dynamic biomolecules to counter plant biotic and abiotic stresses. Int. J. Mol. Sci. 20, 5321. doi: 10.3390/ijms20215321 31731530 PMC6862505

[B50] Vogel J. T. Liu W. Olhoft P. Crafts-Brandner S. J. Pennycooke J. C. Christiansen N. (2021). Soybean yield formation physiology–a foundation for precision breedingbased improvement. Front. Plant Sci. 12, 719706. doi: 10.3389/fpls.2021.719706 34868106 PMC8634342

[B51] Wang Y. Huang M. Gao P. Chen H. Zheng Y. Yang C.. (2021). Expression of heat shock protein ('HSP') genes and antioxidant enzyme genes in hybrid rice II YOU 838 during heat stress. Aust. J. Crop Sci. 15 (9), 37–42. doi: 10.21475/ajcs.21.15.09.sp-4

[B52] Xu Z. Jiang Y. Jia B. Zhou G. (2016). Elevated-CO2 response of stomata and its dependence on environmental factors. Front. Plant Sci. 7, 657. doi: 10.3389/fpls.2016.00657 27242858 PMC4865672

[B53] Yang L. Song W. Xu C. Sapey E. Jiang D. Wu C. (2023). Effects of high night temperature on soybean yield and compositions. Front. Plant Sci. 14, 1065604. doi: 10.3389/fpls.2023.1065604 36890900 PMC9987466

[B54] Zhang H. Y. Hou Z. H. Zhang Y. Li Z. Y. Chen J. Zhou Y. B.. (2022). A soybean EF-Tu family protein GmEF8, an interactor of GmCBL1, enhances drought and heat tolerance in transgenic Arabidopsis and soybean. Int. J. Biol. Macromol. 205, 462–472. doi: 10.1016/j.ijbiomac.2022.01.165 35122805

[B55] Zhang Y. Zhang B. Yang T. Zhang J. Liu B. Zhan X.. (2020). The GAMYB-like gene SlMYB33 mediates flowering and pollen development in tomato. Hortic. Res. 7, 133. doi: 10.1038/s41438-020-00366-1 32922805 PMC7459326

[B56] Zhao C. Liu B. Piao S. Wang X. Lobell D. B. Huang Y.. (2017). Temperature increase reduces global yields of major crops in four independent estimates. PNAS 114 (35), 9326–9331. doi: 10.1073/pnas.1701762114 28811375 PMC5584412

[B57] Zhao L. Wang Z. Lu Q. Wang P. Li Y. Lv Q.. (2013). Overexpression of a GmGBP1 ortholog of soybean enhances the responses to flowering, stem elongation and heat tolerance in transgenic tobaccos. Plant Mol. Biol. 82 (3), 279–299. doi: 10.1007/s11103-013-0062-z 23636865

[B58] Zhou L. Wang C. Liu R. Han Q. Vandeleur R. K. Du J.. (2014). Constitutive overexpression of soybean plasma membrane intrinsic protein GmPIP1; 6 confers salt tolerance. BMC Plant Biol. 14 (1), 1–13. 10.1186/1471-2229-14-181 PMC4105146 24998596

